# BMI and Physical Activity, Military-Aged U.S. Population 2015–2020

**DOI:** 10.1016/j.amepre.2022.08.008

**Published:** 2022-09-23

**Authors:** Bryant J. Webber, Daniel B. Bornstein, Patricia A. Deuster, Francis G. O’Connor, Sohyun Park, Kenneth M. Rose, Geoffrey P. Whitfield

**Affiliations:** 1 Division of Nutrition, Physical Activity, and Obesity, National Center for Chronic Disease Prevention and Health Promotion, Centers for Disease Control and Prevention, Atlanta, Georgia;; 2 Epidemic Intelligence Service, Centers for Disease Control and Prevention, Atlanta, Georgia;; 3 Air Force Institute of Technology, Wright-Patterson Air Force Base, Dayton, Ohio;; 4 DBornsteinSolutions, LLC, Norwich, Vermont;; 5 Consortium for Health and Military Performance, Department of Military and Emergency Medicine, Uniformed Services University of the Health Sciences, Bethesda, Maryland

## Abstract

**Introduction:**

Obesity and physical inactivity are considered possible U.S. national security threats because of their impact on military recruitment. The objectives of this study were to estimate the prevalence of (1) BMI eligibility for military entrance, (2) adequate physical activity participation among the BMI-eligible population, and (3) combined BMI eligibility and adequate physical activity.

**Methods:**

This cross-sectional study of nonpregnant, military-aged civilians (aged 17–42 years) used objectively measured weight and height data and self-reported aerobic physical activity data from the 2015–2020 National Health and Nutrition Examination Survey. *BMI eligibility* was defined as 19.0–27.5 kg/m^2^, per Department of Defense regulation. *Adequate physical activity for entering initial military training* was defined as ≥300 minutes/week of equivalent moderate-intensity aerobic physical activity from all domains, approximating U.S. Army guidance. Participants meeting both definitions were further classified as eligible and active. Analyses were conducted in 2021–2022.

**Results:**

Of military-aged participants (unweighted *n*=5,964), 47.3% were eligible by BMI. Among BMI-eligible participants, 72.5% reported adequate physical activity. Taken together, 34.3% were both eligible and active. The prevalence of eligible and active status was higher among males, persons who were younger and non-Hispanic White, college graduates, and those with higher family income than among their counterparts.

**Conclusions:**

Among the military-aged U.S. population, slightly under half were eligible to enter the military on the basis of their BMI, and only 1 in 3 met BMI eligibility and were adequately physically active. Equitable promotion of healthy weight achievement and physical activity participation may improve military preparedness.

## INTRODUCTION

Physical fitness is one of several eligibility criteria for U.S. military enlistment, appointment, or induction.^1^ Body composition is the primary parameter of physical fitness eligibility, and individual military Services may add traditional measures of physical fitness, such as aerobic capacity.^1,2^ For example, U.S. Army recruiters administer a strength and endurance examination to certify that a recruit is ready for the rigors of training and is physically prepared for the predesignated military occupation.^3^ Before graduating from initial military training (IMT), the new soldier must achieve a passing score on the Army Combat Fitness Test.^4^

In a series of reports^5–9^ drawing on published data^10–12^ and internal government documents,^13,14^ a consortium of retired military leaders emphasize preparedness shortcomings across the U.S. young adult population. According to the latest report, released in 2018,^9^ 31% of the population aged 17–24 years were ineligible to serve because of obesity. Echoing concerns raised in the 1950s,^15^ these leaders and others^16,17^ have characterized widespread obesity and physical inactivity as threats to national security, which jeopardize retention of current service members and recruitment of new service members.

The well-documented societal trends in health-related behaviors and obesity^18–20^ challenge military recruitment efforts. In 2017–2018, obesity prevalence in the U.S. among persons aged 12–19 and 20–39 years was 21%^18^ and 40%,^21^ respectively—up from 18%^18^ and 31%,^19^ respectively, in 2007–2008. Health-related behaviors of U.S. high-school students have mostly trended in the less healthful direction during this time period. Although sugar-sweetened beverage consumption has declined, it remains high,^22^ and youth dietary patterns have shifted toward more ultraprocessed foods and fewer vegetables.^23^ Adolescent physical activity has also decreased.^22,24^ Meanwhile, more adults aged 18–44 years are meeting the combined aerobic and muscle-strengthening physical activity guidelines in leisure time, but the 2018 prevalence remained below 25%.^25^

The impact of these trends on military preparedness is unknown. The most recently published manuscript on the topic used 2007–2008 data.^10^ Moreover, previous preparedness studies did not examine physical activity.^10–14^ Physical activity before military entrance is inversely associated with costly discharge from IMT across the Services, including in the U.S. Army,^26^ Marine Corps,^27^ Navy,^28^ and Air Force.^29^ It is also directly associated with physical fitness,^30^ which predicts training-related musculoskeletal injury^31–38^ and training discharge.^38–41^

The objectives of this study were to estimate the percentage of the military-aged U.S. civilian population who were eligible for military entrance by BMI, the percentage of BMI-eligible who report an adequate physical activity level for IMT, and the combined percentage who were both BMI eligible and adequately physically active. These are vital datapoints for understanding the pool of potential military recruits and for scoping obesity prevention and physical activity promotion efforts.

## METHODS

### Study Sample

All data were acquired from the National Health and Nutrition Examination Survey (NHANES), a multistage probability survey of a nationally representative sample of the civilian, non-institutionalized U.S. population. To produce nationally reliable statistics, NHANES visits 15 counties annually and oversamples Black and Hispanic persons. Detailed methodology is available else-where.^42^ This cross-sectional study incorporated the 2015–2016 cycle and the 2017–2020 (prepandemic) cycle, in which the National Center for Health Statistics (NCHS) merged data from 2017 to 2018 with data from January 2019 to March 2020, when field operations were suspended because of coronavirus disease 2019 (COVID-19).^43^ NHANES is approved by the NCHS Research Ethics Review Board. All participants provided informed consent. Response rates of the examined samples in the 2015–2016 and 2017–2020 cycles were 58.7% and 46.9%, respectively.^44^ The sample was restricted to nonpregnant participants aged 17–42 years, the permissible age range for regular enlistment, appointment, or induction in the U.S. military,^1^ and participants with missing anthropometric and physical activity data were excluded.

In both NHANES cycles, participants reported their gender, age, race, Hispanic origin, the highest level of education, and family income in the previous calendar year. Age was categorized into 4 groups: 17–24, 25–29, 30–34, and 35–42 years. The youngest group, which represents 88% of enlisted military applicants,^45^ facilitates comparison with a recent report.^9^ Race and Hispanic origin were merged by NCHS into a single variable and consolidated into 4 groups: non-Hispanic White, non-Hispanic Black, Hispanic, and non-Hispanic other. Education was categorized into 3 groups (less than high school, some college, and college graduate) and was restricted to persons aged ≥20 years, ages for which NHANES collects education levels for adults.^46^ For many young adults, current educational achievement may not reflect final educational achievement. To account for this, a sensitivity analysis was conducted by restricting to participants aged 25–42 years. NCHS provided the poverty income ratio, the ratio of the reported family income to the Department of Health and Human Services poverty guideline during the respective survey year. Replicating the methodology of a recent study,^47^ poverty income ratio was stratified as low (<1.5), moderate (1.5–4.0), and high (>4.0). Participants missing a stratification variable value were excluded from the analysis of that variable.

### Measures

#### Eligible BMI

Eligible BMI was defined as 19.0–27.5 kg/m^2^, a range that closely approximates a military-wide Department of Defense entrance standard.^48^ This does not exactly demarcate the entrance standard for all applicants because individual Services may issue waivers for candidates not meeting standards,^49^ may use non-BMI measures to assess body composition, and may establish more stringent standards (but not beyond <25.0 kg/m^2^).^48^ Using measured weight and height data in the NHANES examination module, BMI was calculated as the weight in kilograms divided by the square of height in meters and rounded to the nearest tenth place.^50^

#### Adequate physical activity

Adequate physical activity was defined as reporting at least 300 minutes/week of equivalent moderate-intensity aerobic physical activity from all domains (i.e., leisure time, transportation, and, in NHANES, combined occupational and household). This approximates the amount of physical activity—60 minutes per day, 4–5 days per week—recommended by the U.S. Army Future Soldier Program to prepare incoming recruits for IMT.^3^ It also corresponds to the high aerobic guideline of the *Physical Activity Guidelines for Americans*, second edition.^51^ However, by necessity and by design, this definition does not strictly reflect the U.S. Army recommendation because the example physical activities listed in the NHANES questionnaire^52^ do not perfectly overlap with the suggested workouts in the Future Soldier Program.^3^

All-domain physical activity over the previous 30 days was ascertained from a series of 15 questions in NHANES (Appendix Table 1, available online).^52^ To calculate equivalent moderate-intensity duration, vigorous physical activity minutes were multiplied by 2 and added to moderate physical activity minutes.^51^ Beginning in 2017, NHANES restricted the detailed physical activity question set to adults, asking younger participants how many days in the previous week they achieved at least 60 minutes of moderate-to-vigorous intensity physical activity. Accordingly, for those aged 17 years in the 2017–2020 cycle (unweighted *n*=240), *adequate physical activity* was defined as ≥60 minutes per day of moderate-to-vigorous physical activity, the youth recommendation in the *Physical Activity Guidelines for Americans*, second edition.^51^ A sensitivity analysis was performed by excluding participants aged 17 years in the 2017–2020 cycle.

#### Eligible and active

Eligible and active was defined as having an eligible BMI of 19.0–27.5 kg/m^2^ and reporting an adequate physical activity level of at least 300 minutes/week of equivalent moderate-intensity activity. Given the importance of young adults to the military, eligible and active prevalence was calculated by sociodemographic category within the subpopulation aged 17–24 years.

### Statistical Analysis

Data from the 2015–2016 and 2017–2020 NHANES cycles were merged. To account for the 5.2-year surveillance period, the 2015–2016 survey weights were multiplied by 2/5.2, and the 2017–2020 survey weights were multiplied by 3.2/5.2, as recommended by NCHS.^46^ Survey weights from the examination module were used because there were fewer observations for examinations than for interviews in both cycles.

Prevalences of eligible BMI, adequate physical activity, and eligible and active status were calculated for the population and for subpopulations by gender, age category, race/ethnicity, education, and family income. Prevalence within subpopulations was compared using the Satterthwaite-adjusted *F*-test, with significance established at *α* of 0.05. Prevalence estimates and 95% CI were obtained using Taylor Series Linearization methods. Estimates based on low sample sizes or wide Korn–Graubard CI were suppressed, according to NCHS data presentation standards.^53^ To account for the complex survey design, analyses were conducted in SAS-callable SUDAAN, release 11.0.0 (RTI International, Research Triangle Park, NC). The STROBE statement on cross-sectional studies was used to develop this report.^54^

## RESULTS

After excluding eligible participants missing measured weight and height values (unweighted *n*=45) or complete physical activity values (unweighted *n*=75), the unweighted analytic sample size was 5,964. More than half of the weighted sample was male (51.4%) and non-Hispanic White (55.2%). By sociodemographic category, pluralities were aged 17–24 years (29.8%), had completed high school or less (35.2%), and reported a moderate family income (39.0%) (Table 1).

Weighted prevalence of an eligible BMI was 47.3% (95% CI=44.6, 49.9), and that of an ineligible BMI was 52.7% (95% CI=50.1, 55.4), with BMI <19.0 kg/m^2^ comprising 3.9% and BMI >27.5 kg/m^2^ comprising 48.9% of the population. BMI eligibility was similar among males (47.2%) and females (47.3%) (*p*=0.994) but varied significantly by age (*p*<0.001), race/ethnicity (*p*<0.001), education (*p*<0.001), and income (*p*=0.030). Within the population aged 17–24 years, 55.4% were BMI eligible, 7.3% had a low BMI, and 37.3% had a high BMI (Table 2).

Among those with an eligible BMI (unweighted *n*=2,812), weighted prevalence of adequate physical activity was 72.5% (95% CI=70.4, 74.5), and that of inadequate physical activity was 27.5% (95% CI=25.5, 29.6). Adequate physical activity was higher among males than among females (*p*=0.001) and varied significantly by race/ethnicity (*p*<0.001); it was similar by age (*p*=0.186), education (*p*=0.523), and income (*p*=0.553). Among those ineligible owing to low and high BMI, respective weighted prevalence of adequate physical activity was 63.7% and 63.5%. Irrespective of BMI, 67.7% of the population was adequately physically active (Table 3).

Overall, 34.3% (95% CI=31.9, 36.7) of participants were eligible and active. The prevalence was higher among males than among females (*p*=0.047), was lower with older age (*p*<0.001), and varied significantly by race/ethnicity (*p*<0.001), education (*p*=0.001), and income (*p*=0.036). Among those aged 17–24 years, 41.1% were eligible and active (Table 4). Within the young adult population, prevalence differed by race/ethnicity (Appendix Table 2, available online).

Among participants aged 25–42 years (unweighted *n*=3,918), the most common education level was college graduate (weighted prevalence=36.4%). This slightly changed the outcome prevalence: 37.9% of college graduates in this age group were eligible and active, compared with 39.8% in the full sample (Appendix Table 3, available online). Excluding the subset of participants aged 17 years in the 2017–2020 NHANES cycle, adequate physical activity prevalence was 69.3%, and eligible and active prevalence was 34.9%—both slightly higher than in the full sample (Appendix Table 4, available online).

## DISCUSSION

Of the military-aged U.S. civilian population, 47.3% had an eligible BMI for military entrance. Among those eligible by BMI, 72.5% engaged in an adequate level of physical activity. Accounting for both BMI and physical activity, only 34.3% were BMI eligible and active. This is the first military preparedness study incorporating both anthropometric and physical activity metrics.

The estimate for body composition ineligibility in this study exceeds those in previous studies. Applying the 2-stage screening method used by the U.S. Army then,^55^ Cawley and Maclean documented a dramatic rise in the population aged 17–42 years who did not meet body composition standards: whereas 4% of males and 16% of females exceeded standards in NHANES I (conducted from 1971 to 1975), 12% and 35% exceeded the standards in the 2007–2008 NHANES cycle.^10^ In a distinct analysis using 2001–2004 NHANES data and applying Service-specific standards in use then, excessive BMI ineligibility of the population aged 17–42 years ranged from 31% for the U.S. Marine Corps to 40% for the U.S. Air Force.^11^ Methodologic differences—some of which reflect changes in how the Department of Defense assesses body composition—prevent direct comparisons between studies. However, the upward trend in ineligibility running through these 3 studies mirrors the increase in population prevalence of obesity.^18,56^

Similar to these studies,^10,11^ the current analysis includes the entire age-eligible U.S. population. However, reports on military preparedness often focus on young adults.^5–9,12,17^ This is understandable given their disproportionate representation in the enlisted active component of the U.S. military: individuals aged 17–24 years account for 88% of applicants^45^ and 47% of current service members.^57^ A recent report asserts that 31% of the U.S. population aged 17–24 years would be disqualified from military service, if they chose to join, because of obesity.^9^ For the same age group in this study, 37% were ineligible on the basis of high BMI, and 59% were not eligible and active. Some fraction of the eligible cohort would not meet other military entrance requirements (e.g., by virtue of a criminal record^1^ or medically disqualifying condition^49^).

This study also draws attention to the military preparedness repercussions of the inequitable distribution of unhealthy weight and inadequate physical activity. Fewer than 30% of the non-Hispanic Black and Hispanic populations were eligible and active, and prevalence was lower in noncollege graduates than in their peers. Addressing the root causes of health disparities is an established public health priority^58^ that may benefit the U.S. military by expanding enlistment opportunities within underserved populations. The active duty force (*n*=1.33 million) has lower proportions of persons identifying as non-Hispanic White (55.6% vs 62.1%) and who hold a college degree (22.5% vs 40.6%) than the nation’s civilian labor force (*n*=162 million).^59,60^ This diverse workforce positions the U.S. military, in addition to its national defense function, as an important institution for upward mobility.

Equitable strategies to improve nutrition and physical activity can reduce the disparities identified in this study while increasing overall preparedness. Many of these strategies have been synthesized in U.S. Surgeon General reports that endorse access to healthy foods^61^ and to safe and convenient places for walking,^62^ with an emphasis on resource-limited communities.^63^ Evidence-based strategies are also promoted in Active People, Healthy Nation, an initiative by the Centers for Disease Control and Prevention to increase physical activity through individual and community supports.^64^ Municipal leaders can use the Centers for Disease Control and Prevention’s *Active Communities Tool* to enhance street design and connectivity, expand pedestrian and bicycle infrastructure, and extend access to parks and recreational facilities.^65^

As others have asserted,^6,9,16,66^ the military recruitment challenges of tomorrow may be best addressed today by encouraging healthy diet and physical activity among children and adolescents—habits which often continue into adulthood and may protect against obesity later in life.^67^ Challenges abound in the U.S., including racial and ethnic disparities in the prevalence of childhood obesity and inadequate physical activity^24^ and reduced access to nutritious food^61,68^ and safe spaces for physical activity^62,69^ in lower-income communities. These multifaceted problems require engagement by parents, schools, communities, industry, and healthcare providers. The U.S. Preventive Services Task Force recommends universal obesity screening for children aged ≥6 years and referring those with obesity to comprehensive behavioral interventions.^70^ The Community Preventive Services Task Force outlines several evidence-based interventions to prevent and reduce obesity^71^ and to promote physical activity^72^ among youth. Early life solutions include devices to track and reduce recreational screen time^73^; traffic-mitigation strategies to increase walking to and from school^74^; and increased opportunities for physical activity before, during, and after school.^75,76^

### Limitations

The strength of this study is its large, nationally representative sample, but its findings are subject to at least 5 limitations. First, the Service-agnostic BMI range may not strictly correspond to eligibility for military entrance. It does not account for medical waivers, more stringent Service-specific cutoffs (e.g., a BMI threshold of 25.0 kg/m^2^ for young female U.S. Army candidates^77^), or alternative screening techniques for body composition. However, in the population aged 20–39 years, BMI is highly correlated with other modalities that estimate body fat percentage.^78^ Second, physical activity participation was self-reported and limited to bouts of at least 10 minutes. Despite susceptibility to recall and social desirability biases, self-reported pre-entrance physical activity has been strongly associated with multiple performance measures among military recruits.^28,30^ Research also suggests that failure to capture shorter-activity bouts would not dramatically alter these findings.^79^ Third, educational achievement is not static in this cohort of mostly young adults and was not collected for those aged 17–19 years in the later NHANES. Fourth, assessment of physical activity for participants aged 17 years changed between NHANES cycles. To address these latter 2 issues, sensitivity analyses were conducted to provide outcome prevalence under different restriction parameters.

Finally, physical activity participation does not precisely reflect physical fitness.^3^ NHANES was selected because it contains objectively measured BMI and all domains of aerobic physical activity. Including transportation, occupational, and household physical activity may capture individuals who are aerobically prepared for IMT by virtue of physically demanding commutes or occupations but who would be miscategorized by surveillance systems that limit to the leisure-time domain.^80^ Although both endurance and muscular fitness are required for military training, no surveillance system includes all pertinent information. If the study were repeated with leisure-time aerobic and muscle-strengthening activity data in the National Health Interview Survey, the prevalence of adequate physical activity may be even lower. In 2018, just 34% of participants aged 18–24 years reported ≥150 minutes/week of equivalent moderate-intensity physical activity and ≥2 episodes/week of muscle-strengthening activity.^25^

## CONCLUSIONS

Between 2015 and 2020, nearly half of the military-aged U.S. population was eligible to enter the military on the basis of their BMI, and only 1 in 3 was both BMI eligible and adequately active. This is the first military preparedness study that incorporates anthropometric-based eligibility and physical activity. Improving the health of this population and thereby expanding the pool of eligible military applicants require commitment from sectors beyond public health. Upstream efforts can help, including behavioral education, built environment, and policy approaches that promote healthy eating and physical activity. Continued innovation and collaboration may address the root causes of the health inequities reported in this paper.^58^ In 1960, then President-elect John F. Kennedy described our increasing lack of physical fitness as a menace to our security and summoned a national response.^81^ Six decades later, this study contributes new evidence to the ongoing dialogue concerning population health and national security.

## Supplementary Material

Supplementary Material

## Figures and Tables

**Table 1.** Participant Characteristics of the Military-Aged U.S. Population, NHANES 2015–2020

Variables	Population, *n* (weighted %)
Gender	
Male	2,927 (51.4)
Female	3,037 (48.6)
Age, years	
17–24	2,045 (29.8)
25–29	1,094 (20.6)
30–34	1,107 (20.1)
35–42	1,718 (29.5)
Race and ethnicity	
NH White	1,752 (55.2)
NH Black	1,435 (12.7)
Hispanic	1,636 (20.9)
NH other^a^	1,141 (11.2)
Education^b^	
High school or less	1,918 (35.2)
Some college	1,677 (32.2)
College graduate	1,318 (32.6)
Family income^c^	
Low	2,064 (28.4)
Moderate	2,039 (39.0)
High	1,151 (32.6)

*Note:* Data are restricted to nonpregnant participants aged 17–42 years with available BMI and physical activity data; unweighted *n*=5,964 for all variables except for education (*n*=4,913) and family income (*n*=5,254).

NH, non-Hispanic; NHANES, National Health and Nutrition Examination Survey.

a Includes persons who identify as non-Hispanic and any of American Indian or Alaska Native, Asian, Native Hawaiian or Pacific Islander, or multiracial.

b Restricted to participants aged 20–42 years.

c Defined by the poverty income ratio: low, <150%; moderate, 150%–400%; high, >400%.

**Table 2.** Eligible and Ineligible BMI Prevalence of the Military-Aged U.S. Population, NHANES 2015–2020

Variables	Eligible BMI (19.0–27.5 kg/m^2^), % (95% CI)	*p*-value^a^	Ineligible BMI			
			<19.0 kg/m^2^, % (95% CI)	*p*-value^a^	>27.5 kg/m^2^, % (95% CI)	*p*-value^a^
Total	47.3 (44.6, 49.9)		3.9 (3.3, 4.6)		48.9 (46.2, 51.5)	
Gender						
Male	47.2 (42.9, 51.6)	0.994	3.1 (2.4, 4.0)	**0.025**	49.6 (45.4, 53.9)	**0.015**
Female	47.3 (44.5, 50.1)		4.7 (3.7, 5.9)		48.0 (45.4, 50.7)	
Age, years						
17–24	55.4 (51.8, 58.9)	**<0.001**	7.3 (5.9, 8.9)	-	37.3 (33.9, 40.9)	**<0.001**
25–29	49.4 (44.4, 54.4)		4.7 (2.9, 7.5)		45.9 (40.6, 51.4)	
30–34	43.7 (39.2, 48.2)		-		54.5 (50.0, 59.0)	
35–42	40.0 (36.9, 43.3)		-		58.6 (55.0, 62.2)	
Race/ethnicity						
NH White	49.4 (45.6, 53.2)	**<0.001**	4.1 (3.1, 5.3)	0.366	46.5 (42.8, 50.3)	**0.043**
NH Black	43.2 (39.8, 46.6)		3.7 (2.4, 5.7)		53.1 (49.8, 56.5)	
Hispanic	39.6 (36.6, 42.7)		3.0 (2.2, 4.3)		57.3 (53.9, 60.7)	
NH other	55.5 (51.4, 59.4)		4.9 (3.6, 6.6)		39.6 (35.8, 43.6)	
Education^b^						
High school or less	42.2 (38.9, 45.6)	**<0.001**	3.6 (2.5, 5.2)	0.905	54.2 (50.7, 57.6)	0.743
Some college	40.4 (36.7, 44.2)		3.4 (2.5, 4.6)		56.2 (52.6, 59.8)	
College graduate	54.2 (49.3, 59.1)		3.2 (1.9, 5.4)		42.6 (37.8, 47.5)	
Family income^c^						
Low	43.8 (41.3, 46.5)	**0.030**	4.9 (3.5, 6.8)	**0.020**	51.3 (48.3, 54.3)	0.071
Moderate	44.4 (41.3, 47.7)		4.5 (3.3, 6.2)		51.0 (47.6, 54.5)	
High	52.2 (45.7, 58.6)		2.1 (1.4, 3.0)		45.8 (39.2, 52.5)	

*Note:* Boldface indicates statistical significance (*p*<0.05).

Values are weighted percentages on the basis of nonpregnant persons aged 17–42 years; unweighted *n*=5,964 for all variables except for education (*n*=4,913) and family income (*n*=5,254). Weighted percentages may not sum to 100% because of rounding. - denotes data suppressed because of small sample size or wide Korn-Graubard CI.

NH, non-Hispanic; NHANES, National Health and Nutrition Examination Survey.

a Based on the Satterthwaite adjusted *F*-test.

b Restricted to participants aged 20–42 years.

c Defined by the poverty income ratio: low, <150%; moderate, 150%–400%; high, >400%.

**Table 3.** Adequate Physical Activity Prevalence by BMI Eligibility of the Military-Aged U.S. Population, NHANES 2015–2020

Variable	Eligible BMI (19.0 −27.5 kg/m^2^), % (95% CI)	Ineligible BMI					Total, % (95% CI)	*p*-value^a^
		*p*-value^a^	<19.0 kg/m^2^, % (95% CI)	*p*-value^a^	>27.5 kg/m^2^, % (95% CI)	*p*-value^a^		
Total	72.5 (70.4, 74.5)		63.7 (56.3, 70.5)		63.5 (60.9, 65.9)		67.7 (66.1, 69.4)	
Gender								
Male	77.8 (74.4, 80.9)	**0.001**	71.0 (56.9, 82.0)	0.128	72.0 (68.7, 75.1)	**<0.001**	74.7 (72.1, 77.2)	**<0.001**
Female	66.9 (62.8, 70.7)		58.5 (50.3, 66.3)		54.1 (50.5, 57.7)		60.4 (58.1, 62.6)	
Age, years								
17–24	74.2 (70.3, 77.8)	0.186	58.2 (49.8, 66.1)	-	66.7 (63.4, 69.9)	**0.032**	70.3 (67.5, 72.9)	**0.003**
25–29	73.6 (68.4, 78.2)		-		66.4 (61.4, 71.1)		70.4 (66.7, 73.9)	
30–34	74.2 (68.2, 79.4)		-		65.3 (59.5, 70.7)		69.3 (65.2, 73.0)	
35–42	67.9 (63.2, 72.3)		-		58.6 (53.8, 63.2)		62.3 (59.0, 65.5)	
Race/ethnicity								
NH White	77.8 (74.5, 80.7)	**<0.001**	72.2 (61.6, 80.7)	-	64.3 (60.1, 68.3)	0.439	71.3 (68.5, 73.9)	**<0.001**
NH Black	67.3 (63.3, 71.0)		63.2 (50.4, 74.3)		63.6 (60.4, 66.7)		65.2 (62.6, 67.7)	
Hispanic	68.6 (63.4, 73.5)		-		63.5 (59.1, 67.7)		65.1 (62.0, 68.1)	
NH other	59.1 (55.0, 63.1)		-		58.4 (51.4, 65.0)		58.2 (53.9, 62.5)	
Education^b^								
High school or less	73.2 (70.0, 76.3)	0.523	73.3 (60.9, 82.8)	-	64.4 (60.4, 68.2)	0.054	68.5 (65.7, 71.1)	0.108
Some college	76.3 (71.5, 80.6)		75.2 (60.6, 85.7)		68.5 (64.6, 72.2)		71.9 (69.0, 74.7)	
College graduate	73.4 (69.1, 77.3)		-		59.2 (51.9, 66.1)		67.0 (63.1, 70.7)	
Family income^c^								
Low	72.4 (68.7, 75.9)	0.553	60.5 (47.0, 72.5)	-	61.2 (57.9, 64.4)	0.213	66.1 (64.0, 68.1)	0.435
Moderate	71.6 (67.9, 75.0)		-		66.3 (62.2, 70.2)		68.8 (65.5, 72.0)
High	73.4 (69.1, 77.3)		-		59.2 (51.9, 66.1)		67.0 (63.1, 70.7)	

*Note:* Boldface indicates statistical significance (*p*<0.05).

Data are weighted percentages on the basis of nonpregnant persons aged 17–42 years; unweighted *n*=5,964 for all variables except for education (*n*=4,913) and family income (*n*=5,254), defined as reporting ≥300 minutes/week of moderate-intensity physical activity or ≥150 minutes/week of vigorous-intensity physical activity or the equivalent combination from all domains (or for those aged 17 years in the 2017–2020 data, reporting ≥60 minutes/day of moderate-intensity physical activity daily). – denotes data suppressed owing to small sample size or wide Korn–Graubard CI.

NH, non-Hispanic; NHANES, National Health and Nutrition Examination Survey; PA, physical activity.

a Based on the Satterthwaite adjusted *F*-test.

b Restricted to participants aged 20–42 years.

c Defined by the poverty income ratio: low, <150%; moderate, 150%–400%; high, >400%.

**Table 4.** Combined Prevalence of Eligible and Active Status of the Military-Aged U.S. Population, NHANES 2015–2020

Variables	Eligible and active,^a^ % (95% CI)	Not eligible and active,^b^ % (95% CI)	*p*-value^c^
Total	34.3 (31.9, 36.7)	65.7 (63.3, 68.1)	
Gender			
Male	36.8 (33.1, 40.6)	63.2 (59.4, 66.9)	**0.047**
Female	31.6 (28.5, 34.9)	68.4 (65.1, 71.5)	
Age, years			
17–24	41.1 (37.1, 45.1)	58.9 (54.9, 62.9)	**<0.001**
25–29	36.3 (31.8, 41.1)	63.7 (58.9, 68.2)	
30–34	32.4 (28.1, 37.0)	67.6 (63.0, 71.9)	
35–42	27.2 (24.3, 30.2)	72.8 (69.8, 75.7)	
Race/ethnicity			
NH White	38.4 (34.8, 42.2)	61.6 (57.8, 65.2)	**<0.001**
NH Black	29.1 (26.1, 32.1)	70.9 (67.9, 73.9)	
Hispanic	27.2 (24.3, 30.3)	72.8 (69.7, 75.7)	
NH other	32.8 (29.8, 35.8)	67.2 (64.2, 70.2)	
Education^d^			
High school or less	30.9 (28.1, 33.9)	69.1 (66.1, 71.9)	**0.001**
Some college	30.8 (27.2, 34.7)	69.2 (65.3, 72.8)	
College graduate	39.8 (35.5, 44.2)	60.2 (55.8, 64.5)	
Family income^e^			
Low	31.8 (29.0, 34.6)	68.2 (65.4, 71.0)	**0.036**
Moderate	31.8 (29.3, 34.5)	68.2 (65.5, 70.7)	
High	38.8 (32.9, 45.0)	61.2 (55.0, 67.1)	

*Note:* Boldface indicates statistical significance (*p*<0.05).

Values are weighted percentages on the basis of nonpregnant persons aged 17–42 years; unweighted *n*=5,964 for all variables except for education (*n*=4,913) and family income (*n*=5,254).

NH, non-Hispanic; NHANES, National Health and Nutrition Examination Survey; PA, physical activity.

a Defined as BMI of 19.0–27.5 kg/m^2^ and reporting ≥300 minutes/week of moderate-intensity PA or ≥150 minutes/week of vigorous-intensity PA or the equivalent combination from all domains (or for those aged 17 years in the 2017–2020 data, reporting ≥60 minutes/day of moderate-intensity PA daily).

b Defined as BMI <19.0 or >27.5 kg/m^2^ or reporting <300 minutes/week of moderate-intensity PA or <150 minutes/week of vigorous-intensity PA or the equivalent combination from all domains (or for those aged 17 years in the 2017–2020 data, not reporting ≥60 minutes/day of moderate-intensity PA daily).

c Based on the Satterthwaite adjusted *F*-test.

d Restricted to participants aged 20–42 years.

e Defined by the poverty income ratio: low, <150%; moderate, 150%–400%; high, >400%.

## References

[1] 32 CFR 66.6: Enlistment, Appointment, and Induction Criteria. Amended July 1, 2022. https://www.ecfr.gov/current/title-32/subtitle-A/chapter-I/subchapter-D/part-66/section-66.6. Accessed July 29, 2022.

[2] Instruction 1304.26: qualification standards for enlistment, appointment, and induction. U.S. Department of Defense. https://www.esd.whs.mil/Portals/54/Documents/DD/issuances/dodi/130426p.pdf?ver=2018-10-26-085822-050. Updated October 26, 2018. Accessed July 29, 2022.

[3] U.S. Department of the Army. FM 7–22: holistic health and fitness. Washington, DC: U.S. Department of the Army; 2020. https://army-pubs.army.mil/epubs/DR_pubs/DR_a/ARN30714-FM_7-22-000-WEB-1.pdf. Published October. Accessed July 29, 2022.

[4] Secretory of the Army Washington. Army Directive 2022–05 (Army Combat Fitness Test). Washington, DC: Secretory of the Army Washington; 2022. https://armypubs.army.mil/epubs/DR_pubs/DR_a/ARN35052-ARMY_DIR_2022-05-000-WEB-1.pdf. Published March 23. Accessed July 29, 2022.

[5] Mission: readiness. Ready, willing, and unable to serve. Strong America. November 5, 2009. https://www.strongnation.org/articles/21-ready-willing-and-unable-to-serve. Accessed July 29, 2022.

[6] Mission: readiness. Too fat to fight. Strong America. April 10, 2010. https://www.strongnation.org/articles/23-too-fat-to-fight. Accessed July 29, 2022.

[7] Mission: readiness. Still too fat to fight. Strong America. September 1, 2012. https://www.strongnation.org/articles/16-still-too-fat-to-fight. Accessed July 29, 2022.

[8] Mission: readiness. Unfit to fight. Strong America. April 30, 2015. https://www.strongnation.org/articles/53-unfit-to-fight. Accessed July 29, 2022.

[9] Mission: readiness. Unhealthy and unprepared. Strong America. October 10, 2018. https://www.strongnation.org/articles/737-unhealthy-and-unprepared. Accessed July 29, 2022.

[10] Cawley J, Maclean JC. Unfit for service: the implications of rising obesity for U.S. military recruitment. Health Econ. 2012;21(11):1348–1366. 10.1002/hec.1794. 21971919

[11] Yamane GK. Obesity in civilian adults: potential impact on eligibility for U.S. military enlistment. Mil Med. 2007;172(11):1160–1165. 10.7205/MILMED.172.11.1160. 18062389

[12] Nolte R, Franckowiak SC, Crespo CJ, Andersen RE. U.S. military weight standards: what percentage of U.S. young adults meet the current standards? Am J Med. 2002;113(6):486–490. 10.1016/S0002-9343(02)01268-8. 12427498

[13] U.S. Department of Defense. Office of Under Secretary of defense for personnel and readiness, joint advertising market research and studies. Qualified military available. October 2013.

[14] U.S. Department of Defense. Office of Under Secretary of defense for personnel and readiness, joint advertising market research and studies. Qualified military available. October 2017.

[15] The federal government takes on physical fitness. John F. Kennedy Presidential Library and Museum. https://www.jfklibrary.org/learn/about-jfk/jfk-in-history/physical-fitness. Updated November 7, 2018. Accessed July 29, 2022.

[16] Tompkins E. Obesity in the United States and effects on military recruiting. Washington, DC: Congressional Research Service; 2020. https://sgp.fas.org/crs/natsec/IF11708.pdf. Published December 22. Accessed July 29, 2022.

[17] Centers for Disease Control and Prevention. Unfit to serve: obesity is impacting national security. Atlanta, GA: Centers for Disease Control and Prevention; 2019. https://stacks.cdc.gov/view/cdc/77062. Published March 28. Accessed July 29, 2022.

[18] Fryar CD, Carroll MD, Afful J. Prevalence of overweight, obesity, and severe obesity among children and adolescents aged 2–19 years: United States, 1963–1965 through 2017–2018. Atlanta, GA: Centers for Disease Control and Prevention; 2020. https://www.google.com/url?sa=t&rct=j&q=&esrc=s&source=web&cd=&cad=rja&uact=8&ve-d=2ahUKEwj8yNn8wvb5AhUBfnAKHS9hBwIQFnoECA8QA-Q&url=https%3A%2F%2Fwww.cdc.gov%2Fnchs%2Fdata%2Fhestat%2Fobesity-child-17-18%2Fobesity-child.htm&usg=AOv-Vaw20vkknS2o7-coLhihdL-1O. Published. Accessed July 29, 2022.

[19] Hales CM, Fryar CD, Carroll MD, Freedman DS, Ogden CL. Trends in obesity and severe obesity prevalence in U.S. youth and adults by sex and age, 2007–2008 to 2015–2016. JAMA. 2018;319(16):1723–1725. 10.1001/jama.2018.3060. 29570750 PMC5876828

[20] Wang Y, Beydoun MA, Min J, Xue H, Kaminsky LA, Cheskin LJ. Has the prevalence of overweight, obesity and central obesity levelled off in the United States? Trends, patterns, disparities, and future projections for the obesity epidemic. Int J Epidemiol. 2020;49(3):810–823. 10.1093/ije/dyz273. 32016289 PMC7394965

[21] Hales CM, Carroll MD, Fryar CD, Ogden CL. Prevalence of obesity and severe obesity among adults: United States, 2017–2018. NCHS Data Brief. 2020;360(360):1–8. https://www.cdc.gov/nchs/data/databriefs/db360-h.pdf. Accessed June 29, 2022. 32487284

[22] Merlo CL, Jones SE, Michael SL, Dietary and physical activity behaviors among high school students - Youth Risk Behavior Survey, United States, 2019. MMWR Suppl. 2020;69(1):64–76. 10.15585/mmwr.su6901a8. 32817612 PMC7440200

[23] Wang L, Martínez Steele E, Du M, Trends in consumption of ultraprocessed foods among U.S. youths aged 2–19 years, 1999–2018. JAMA. 2021;326(6):519–530. 10.1001/jama.2021.10238. 34374722 PMC8356071

[24] Chen TJ, Watson KB, Michael SL, Carlson SA. Sex-stratified trends in meeting physical activity guidelines, participating in sports, and attending physical education among U.S. adolescents, Youth Risk Behavior survey 2009–2019. J Phys Act Health. 2021;18(suppl 1): S102–S113. 10.1123/jpah.2021-0263. 34465644 PMC10035360

[25] Hyde ET, Whitfield GP, Omura JD, Fulton JE, Carlson SA. Trends in meeting the physical activity guidelines: muscle-strengthening alone and combined with aerobic activity, United States, 1998–2018. J Phys Act Health. 2021;18(suppl 1):S37–S44. 10.1123/jpah.2021-0077. 34465652 PMC11000248

[26] Swedler DI, Knapik JJ, Williams KW, Grier TL, Jones BH. Risk factors for medical discharge from United States Army Basic Combat Training. Mil Med. 2011;176(10):1104–1110. 10.7205/MILMED-D-10-00451. 22128643

[27] Reis JP, Trone DW, Macera CA, Rauh MJ. Factors associated with discharge during marine corps basic training. Mil Med. 2007;172(9):936–941. 10.7205/MILMED.172.9.936. 17937356

[28] Trone DW, Cipriani DJ, Raman R, Wingard DL, Shaffer RA, Macera CA. The association of self-reported measures with poor training out-comes among male and female U.S. Navy recruits. Mil Med. 2013;178 (1):43–49. 10.7205/MILMED-D-12-00271. 23356118

[29] Talcott GW, Haddock CK, Klesges RC, Lando H, Fiedler E. Prevalence and predictors of discharge in United States Air Force basic military training. Mil Med. 1999;164(4):269–274. 10.1093/milmed/164.4.269. 10226453

[30] Gubata ME, Cowan DN, Bedno SA, Urban N, Niebuhr DW. Self-reported physical activity and preaccession fitness testing in U.S. Army applicants. Mil Med. 2011;176(8):922–925. 10.7205/MILMED-D-10-00308. 21882783

[31] Bedno SA, Cowan DN, Urban N, Niebuhr DW. Effect of pre-accession physical fitness on training injuries among U.S. army recruits. Work. 2013;44(4):509–515. 10.3233/WOR-2012-1355. 22927579

[32] Krauss MR, Garvin NU, Boivin MR, Cowan DN. Excess stress fractures, musculoskeletal injuries, and health care utilization among unfit and overweight female army trainees. Am J Sports Med. 2017;45 (2):311–316. 10.1177/0363546516675862. 27881384

[33] Bornstein DB, Grieve GL, Clennin MN, Which U.S. states pose the greatest threats to military readiness and public health? Public health policy implications for a cross-sectional investigation of cardio-respiratory fitness, body mass index, and injuries among U.S. Army recruits. J Public Health Manag Pract. 2019;25(1):36–44. 10.1097/PHH.0000000000000778. 29319585

[34] Sefton JM, Lohse KR, McAdam JS. Prediction of injuries and injury types in army basic training, infantry, armor, and cavalry trainees using a common fitness screen. J Athl Train. 2016;51(11):849–857. 10.4085/1062-6050-51.9.09. 28068160 PMC5224725

[35] Jones BH, Hauret KG, Dye SK, Impact of physical fitness and body composition on injury risk among active young adults: a study of army trainees. J Sci Med Sport. 2017;20(suppl 4):S17–S22. 10.1016/j.jsams.2017.09.015. 28993131

[36] Müller-Schilling L, Gundlach N, Böckelmann I, Sammito S. Physical fitness as a risk factor for injuries and excessive stress symptoms during basic military training. Int Arch Occup Environ Health. 2019;92(6):837–841. 10.1007/s00420-019-01423-6. 30895369

[37] Sharma J, Golby J, Greeves J, Spears IR. Biomechanical and lifestyle risk factors for medial tibia stress syndrome in army recruits: a prospective study. Gait Posture. 2011;33(3):361–365. 10.1016/j.gaitpost.2010.12.002. 21247766

[38] Orr RM, Cohen BS, Allison SC, Bulathsinhala L, Zambraski EJ, Jaffrey M. Models to predict injury, physical fitness failure and attrition in recruit training: a retrospective cohort study. Mil Med Res. 2020;7 (1):26. 10.1186/s40779-020-00260-w. 32493512 PMC7271478

[39] Nye NS, Pawlak MT, Webber BJ, Tchandja JN, Milner MR. Description and rate of musculoskeletal injuries in air force basic military trainees, 2012–2014. J Athl Train. 2016;51(11):858–865. 10.4085/1062-6050-51.10.10. 28068163 PMC5224726

[40] Dimitriou L, Lockey J, Castell L. Is baseline aerobic fitness associated with illness and attrition rate in military training? J R Army Med Corps. 2017;163(1):39–47. 10.1136/jramc-2015-000608. 26929111

[41] Niebuhr DW, Scott CT, Powers TE, Assessment of recruit motivation and strength study: preaccession physical fitness assessment predicts early attrition. Mil Med. 2008;173(6):555–562. 10.7205/MILMED.173.6.555. 18595419

[42] About the National Health and Nutrition Examination Survey. National Center for Health Statistics, Centers for Disease Control and Prevention. https://www.cdc.gov/nchs/nhanes/about_nhanes.htm. Updated September 15, 2017. Accessed July 29, 2022.

[43] NHANES questionnaires, datasets, and related documentation. National Center for Health Statistics, Centers for Disease Control and Prevention. https://wwwn.cdc.gov/nchs/nhanes/Default.aspx. Updated June 15, 2022. Accessed July 29, 2022.

[44] NHANES response rates. National Center for Health Statistics, Centers for Disease Control and Prevention. https://wwwn.cdc.gov/nchs/nhanes/responserates.aspx. Updated July 7, 2022. Accessed July 29, 2022.

[45] U.S. Department of Defense. Appendix A: active component applicant tables. Washington, DC: U.S. Department of Defense.; 2021. https://www.cna.org/pop-rep/2019/appendixa/appendixa.pdf. Published November. Accessed July 29, 2022.

[46] NHANES analytic guidance and brief overview for the 2017-March 2020 pre-pandemic data files. National Center for Health Statistics, Centers for Disease Control and Prevention. https://wwwn.cdc.gov/nchs/nhanes/continuousnhanes/overviewbrief.aspx?cycle=2017-2020. Updated June 22, 2021. Accessed July 29, 2022.

[47] Watson KB, Whitfield G, Chen TJ, Hyde ET, Omura JD. Trends in aerobic and muscle-strengthening physical activity by race/ethnicity across income levels among U.S. adults, 1998–2018. J Phys Act Health. 2021;18(suppl 1):S45–S52. 10.1123/jpah.2021-0260. 34465650 PMC8743873

[48] U.S. Department of Defense. Instruction 1308.3: DoD physical fitness and body fat programs procedures. Washington, DC: U.S. Department of Defense; 2022. https://www.esd.whs.mil/Portals/54/Documents/DD/issuances/dodi/130803p.pdf. Published March 10. Accessed July 29, 2022.

[49] U.S. Department of Defense. Instruction 6130.03, volume 1. Medical standards for military service: appointment, enlistment, or induction. Washington, DC: U.S: Department of Defense; 2022. https://www.esd.whs.mil/Portals/54/Documents/DD/issuances/dodi/613003_v1p.PDF. Published June 6. Accessed July 29, 2022.

[50] National Center for Health Statistics. NHANES anthropometry procedures manual. Atlanta, GA: Centers for Disease Control and Prevention; January 2017. https://wwwn.cdc.gov/nchs/data/nhanes/2017-2018/manuals/2017_Anthropometry_Procedures_Manual.pdf. Published. Accessed July 29, 2022.

[51] HHS. Physical Activity Guidelines for Americans. 2nd ed. Washington, DC: HHS; 2018. https://health.gov/sites/default/files/2019-09/Physical_Activity_Guidelines_2nd_edition.pdf. Accessed June 29, 2022.

[52] Physical activity and physical fitness questionnaire. National Center for Health Statistics. http://www.cdc.gov/nchs/data/nhanes/spq-pa.pdf. Updated January 26, 2016. Accessed July 29, 2022.

[53] Parker JD, Talih M, Malec DJ, National Center for Health Statistics data presentation standards for proportions. Vital Health Stat. 2017;2(175):1–22. https://www.cdc.gov/nchs/data/series/sr_02/sr02_175.pdf. Accessed June 29, 2022. 30248016

[54] von Elm E, Altman DG, Egger M, The Strengthening the Reporting of Observational Studies in Epidemiology (STROBE) statement: guidelines for reporting observational studies. Lancet. 2007;370 (9596):1453–1457. 10.1016/S0140-6736(07)61602-X. 18064739

[55] Instruction 1308.3: DoD physical fitness and body fat programs procedures [obsolete]. U.S. Department of Defense. https://biotech.law.lsu.edu/blaw/dodd/corres/html/13083.htm. Updated 2002. Accessed July 29, 2022.

[56] Ogden CL, Fryar CD, Martin CB, Trends in obesity prevalence by race and Hispanic origin [C0]1999–2000 to 2017–2018. JAMA. 2020;324(12):1208–1210. 10.1001/jama.2020.14590. 32857101 PMC7455882

[57] U.S. Department of Defense. Office of Under Secretary of defense for personnel and readiness. Population representation in the military services: fiscal year. Summary report (Appendix B: Active Component Accessions and Force). Washington, DC: U.S. Department of Defense; 2021. https://www.cna.org/pop-rep/2019/appendixb/appendixb.pdf. Published. Accessed July 29, 2022.

[58] Hacker KA, Briss PA. An ounce of prevention is still worth a pound of cure, especially in the time of COVID-19. Prev Chronic Dis. 2021;18: E03. 10.5888/pcd18.200627. 33411667 PMC7845547

[59] U.S. Department of Defense. Office of the deputy assistant Secretary of Defense for military community and family policy; 2020 Demographics Profile of the Military Community. Washington, DC: U.S. Department of Defense; 2021. https://download.militaryonesource.mil/12038/MOS/Reports/2020-demographics-report.pdf. Published. Accessed July 29, 2022.

[60] U.S. Bureau of Labor Statistics. Report 1082: Labor Force Characteristics by Race and Ethnicity. Washington, DC: U.S. Bureau of Labor Statistics; 2018. https://www.bls.gov/opub/reports/race-and-ethnicity/2018/pdf/home.pdf. Published October 2019. Accessed July 29, 2022.

[61] HHS. The Surgeon General’s Vision for a Healthy and Fit Nation. Rockville, MD: Office of the Surgeon General; 2010. https://www.ncbi.nlm.nih.gov/books/NBK44660/. Accessed June 29, 2022. 20669507

[62] HHS. Step it up! The Surgeon General’s Call to Action to Promote Walking and Walkable Communities. Washington, DC: Office of the Surgeon General; 2015. https://www.ncbi.nlm.nih.gov/books/NBK538433/. Accessed June 29, 2022.

[63] Brownson RC, Baker EA, Housemann RA, Brennan LK, Bacak SJ. Environmental and policy determinants of physical activity in the United States. Am J Public Health. 2001;91(12):1995–2003. 10.2105/AJPH.91.12.1995. 11726382 PMC1446921

[64] Schmid TL, Fulton JE, McMahon JM, Devlin HM, Rose KM, Petersen R. Delivering physical activity strategies that work: active People, Healthy NationSM. J Phys Act Health. 2021;18(4):352–356. 10.1123/jpah.2020-0656. 33639612

[65] Centers for Disease Control and Prevention. The active communities tool (ACT): an action planning Guide and assessment modules to improve community built environments to promote physical activity. Atlanta, GA: Centers for Disease Control and Prevention; 2019. https://www.cdc.gov/physicalactivity/community-strategies/active-communities-tool/active-communities-toolkit-form-h.pdf. Published. Accessed July 29, 2022.

[66] Koehlmoos TP, Banaag A, Madsen CK, Adirim T. Child health as a national security issue: obesity and behavioral health conditions among military children. Health Aff (Millwood). 2020;39(10):1719–1727. 10.1377/hlthaff.2020.00712. 33017245

[67] O’ConnorEA, EvansCV, BurdaBU, WalshES, EderM, LozanoP. U. S. Preventive Services Task Force evidence syntheses. screening for obesity and interventions for weight management in children and adolescents: a systematic evidence review for the U.S. Preventive Services Task Force. JAMA. 2017;317(23):2427–2444. 10.1001/jama.2017.0332.28632873

[68] Cooksey-Stowers K, Schwartz MB, Brownell KD. Food swamps predict obesity rates better than food deserts in the United States. Int J Environ Res Public Health. 2017;14(11):1366. 10.3390/ijerph14111366. 29135909 PMC5708005

[69] Burdette HL, Whitaker RC. A national study of neighborhood safety, outdoor play, television viewing, and obesity in preschool children. Pediatrics. 2005;116(3):657–662. 10.1542/peds.2004-2443. 16140705

[70] U.S. Preventive Services Task Force, Grossman DC, Bibbins-Domingo K. Screening for obesity in children and adolescents: U.S. Preventive Services Task Force recommendation statement. JAMA. 2017;317 (23):2417–2426. 10.1001/jama.2017.6803. 28632874

[71] CPSTF findings for obesity. Community Preventive Services Task Force. https://www.thecommunityguide.org/content/task-force-findings-obesity. Updated 2021. Accessed July 29, 2022.

[72.] CPSTF findings for physical activity. Community Preventive Services Task Force. https://www.thecommunityguide.org/content/task-force-findings-physical-activity. Updated April 19, 2022. Accessed July 29, 2022.

[73] Ramsey Buchanan L, Rooks-Peck CR, Finnie RKC, Reducing recreational sedentary screen time: a Community Guide systematic review. Am J Prev Med. 2016;50(3):402–415. 10.1016/j.amepre.2015.09.030. 26897342 PMC9664246

[74] Omura JD, Hyde ET, Watson KB, Sliwa SA, Fulton JE, Carlson SA. Prevalence of children walking to school and related barriers - United States, 2017. Prev Med. 2019;118:191–195. 10.1016/j.ypmed.2018.10.016. 30416098 PMC10917142

[75] Centers for Disease Control and Prevention. Increasing physical education and physical activity: a framework for schools. Atlanta, GA: Centers for Disease Control and Prevention; 2019. https://www.cdc.gov/healthyschools/physicalactivity/pdf/2019_04_25_PE-PA-Frame-work_508tagged.pdf. Published April 25Accessed July 29, 2022.

[76] Masini A, Marini S, Gori D, Leoni E, Rochira A, Dallolio L. Evaluation of school-based interventions of active breaks in primary schools: a systematic review and meta-analysis. J Sci Med Sport. 2020;23(4):377–384. 10.1016/j.jsams.2019.10.008. 31722840

[77] U.S. Department of the Army. Army Regulation: The Army Body Composition Program. Washington, DC: U.S. Department of the Army; 2019. https://armypubs.army.mil/epubs/dr_pubs/dr_a/pdf/web/arn7779_ar600-9_final.pdf. Published July 16. Accessed July 29, 2022.

[78] Flegal KM, Shepherd JA, Looker AC, Comparisons of percentage body fat, body mass index, waist circumference, and waist-stature ratio in adults. Am J Clin Nutr. 2009;89(2):500–508. 10.3945/ajcn.2008.26847. 19116329 PMC2647766

[79] Ussery EN, Watson KB, Carlson SA. The influence of removing the ten-minute bout requirement on national physical activity estimates. Prev Chronic Dis. 2020;17:E19. 10.5888/pcd17.190321. 32105588 PMC7085908

[80] Whitfield GP, Ussery EN, Carlson SA. Combining data from assessments of leisure, occupational, household, and transportation physical activity among U.S. adults, NHANES 2011–2016. Prev Chronic Dis. 2020;17:E117. 10.5888/pcd17.200137. 33006543 PMC7553230

[81] Kennedy JF. Sport at the new frontier: the soft American. Sports Illustrated. 1960;13(26):14–17. https://archive.org/details/Sports-Illustrated-1960-12-26/page/n15/mode/2up. Accessed June 29, 2022.

