# Supplementary material for: BMI and Physical Activity, Military-Aged U.S. Population 2015–2020
Source: Am J Prev Med. Author manuscript; Available in PMC 2023 Jan 30. (PMC9885292; doi:10.1016/j.amepre.2022.08.008)
Supplement: Supplementary Material [file NIHMS1867409-supplement-Supplementary_Material.pdf]

**Appendix Table 1.** Physical Activity Questions from the National Health and Nutrition Examination Survey

| Cycle | Ages | Questions |
|---|---|---|
| 2015–16 | All[a] | 1. Next I am going to ask you about the time {you spend/SP spends} doing different types of physical activity in a typical week. Think first about the time {you spend/he spends/she spends} doing work. Think of work as the things that {you have/he has/she has} to do such as paid or unpaid work, household chores, and yard work. Does {your/SP's} work involve vigorous-intensity activity that causes large increases in breathing or heart rate like carrying or lifting heavy loads, digging or construction work for at least 10 minutes continuously?<br>2. In a typical week, on how many days {do you/does SP} do vigorous-intensity activities as part of {your/his/her} work?<br>3. How much time {do you/does SP} spend doing vigorous-intensity activities at work on a typical day?<br>4. Does {your/SP's} work involve moderate-intensity activity that causes small increases in breathing or heart rate such as brisk walking or carrying light loads for at least 10 minutes continuously?<br>5. In a typical week, on how many days {do you/does SP} do moderate-intensity activities as part of {your/his/her} work?<br>6. How much time {do you/does SP} spend doing moderate-intensity activities at work on a typical day?<br>7. The next questions exclude the physical activity at work that you have already mentioned. Now I would like to ask you about the usual way {you travel/SP travels} to and from places. For example to school, for shopping, to work. In a typical week {do you/does SP} walk or use a bicycle for at least 10 minutes continuously to get to and from places?<br>8. In a typical week, on how many days {do you/does SP} walk or bicycle for at least 10 minutes continuously to get to and from places?<br>9. How much time {do you/does SP} spend walking or bicycling for travel on a typical day?<br>10. The next questions exclude the work and transport activities that you have already mentioned. Now I would like to ask you about sports, fitness and recreational activities. In a typical week {do you/does SP} do any vigorous-intensity sports, fitness, or recreational activities that cause large increases in breathing or heart rate like running or basketball for at least 10 minutes continuously?<br>11. In a typical week, on how many days {do you/does SP} do vigorous-intensity sports, fitness or recreational activities?<br>12. How much time {do you/does SP} spend doing vigorous-intensity sports, fitness or recreational activities on a typical day? |

| | | |
|---|---|---|
| | | 13. In a typical week {do you/does SP} do any moderate-intensity sports, fitness, or recreational activities that cause a small increase in breathing or heart rate such as brisk walking, bicycling, swimming, or volleyball for at least 10 minutes continuously?<br>14. In a typical week, on how many days {do you/does SP} do moderate-intensity sports, fitness or recreational activities?<br>15. How much time {do you/does SP} spend doing moderate-intensity sports, fitness or recreational activities on a typical day? |
| 2017–20 (pre-pandemic) | 18–44 years[b] | Questions are the same as above |
| 2017–20 (pre-pandemic) | 17 years[c] | I'd like to ask you some questions about {your/SP's} activities. During the past 7 days, on how many days {were you/was SP} physically active for a total of at least 60 minutes per day? Add up all the time {you/he/she} spent in any kind of physical activity that increased {your/his/her} heart rate and made {you/him/her} breathe hard some of the time. |

[a]Available at https://wwwn.cdc.gov/Nchs/Nhanes/2015-2016/PAQ_I.htm.
[b]Available at https://wwwn.cdc.gov/Nchs/Nhanes/2017-2018/P_PAQ.htm.
[c]Available at https://wwwn.cdc.gov/Nchs/Nhanes/2017-2018/P_PAQY.htm.

**Appendix Table 2.** Prevalence of "Eligible and Active," Persons Aged 17–24 Years, NHANES 2015–2020

| | **"Eligible and Active"[a]** % (95% CI) | **Not "Eligible and Active"[b]** % (95% CI) | ***p*-value[c]** |
|---|---|---|---|
| Total | 41.1 (37.1, 45.1) | 58.9 (54.9, 62.9) | |
| Gender | | | |
| Male | 44.5 (38.8, 50.2) | 55.5 (49.8, 61.2) | 0.066 |
| Female | 37.4 (32.4, 42.7) | 62.6 (57.3, 67.6) | |
| Race/ethnicity | | | |
| NH White | 45.8 (39.5, 52.2) | 54.2 (47.8, 60.5) | **0.007** |
| NH Black | 39.0 (33.8, 44.4) | 61.0 (55.6, 66.2) | |
| Hispanic | 33.2 (27.7, 39.2) | 66.8 (60.8, 72.3) | |
| NH Other | 37.5 (31.5, 44.0) | 62.5 (56.0, 68.5) | |
| Education[d] | | | |
| High school or less | 38.8 (31.1, 47.2) | 61.2 (52.8, 68.9) | -- |
| Some college | 41.5 (34.2, 49.2) | 58.5 (50.8, 65.8) | |
| College graduate | -- | -- | |
| Family Income[e] | | | |
| Low | 38.4 (32.7, 44.4) | 61.6 (55.6, 67.3) | 0.566 |
| Moderate | 39.2 (35.3, 43.3) | 60.8 (56.7, 64.7) | |
| High | 43.3 (33.0, 54.2) | 56.7 (45.8, 67.0) | |

Note: Boldface indicates statistical significance ($p<0.05$).
Values are weighted percentages based on non-pregnant persons aged 17–24 years; unweighted n=2,045 for all variables except education (n=995) and family income (n=1,772).
NH, non-Hispanic; --, data suppressed due to wide Korn-Graubard confidence interval.
[a]Defined as body mass index 19.0–27.5 kg/m$^2$ and reporting ≥300 minutes/week of moderate-intensity physical activity or ≥150 minutes/week of vigorous-intensity physical activity or the equivalent combination, from all domains (or, for those aged 17 years in the 2017–2020 data, reporting ≥60 minutes/day of moderate-intensity physical activity daily).
[b]Defined as body mass index <19.0 or >27.5 kg/m$^2$ or reporting <300 minutes/week of moderate-intensity physical activity or <150 minutes/week of vigorous-intensity physical activity or the equivalent combination, from all domains (or, for those aged 17 years in the 2017–2020 data, not reporting ≥60 minutes/day of moderate-intensity physical activity daily).
[c]Based on the Satterthwaite adjusted F-test.
[d]Restricted to participants aged 20–24 years.
[e]Defined by the poverty income ratio: low, <150%; moderate, 150–400%; high, >400%.

**Appendix Table 3.** Prevalence of Outcomes by Education Level, Persons Aged ≥25 Years, NHANES 2015–2020

| | **Population** n (weighted %) | **Eligible by Body Mass Index**[a] % (95% CI) | **Adequately Physically Active**[b] % (95% CI) | **"Eligible and Active"**[c] % (95% CI) |
|---|---|---|---|---|
| High school or less | 1,488 (33.5) | 40.3 (36.5, 44.3) | 66.3 (62.9, 69.5) | 28.2 (24.8, 32.0) |
| Some college | 1,255 (30.1) | 36.4 (32.5, 40.5) | 69.5 (66.3, 72.6) | 26.9 (23.2, 31.1) |
| College graduate | 1,175 (36.4) | 53.1 (48.4, 57.9) | 64.7 (60.5, 68.8) | 37.9 (33.6, 42.3) |

Values are weighted percentages based on non-pregnant persons aged 25–42 years; unweighted n=3,918.
[a]Defined as body mass index 19.0–27.5 kg/m$^2$.
[b]Defined as reporting ≥300 minutes/week of moderate-intensity physical activity or ≥150 minutes/week of vigorous-intensity physical activity or the equivalent combination, from all domains.
[c]Defined as body mass index 19.0–27.5 kg/m$^2$ and reporting ≥300 minutes/week of moderate-intensity physical activity or ≥150 minutes/week of vigorous-intensity physical activity or the equivalent combination, from all domains.

**Appendix Table 4.** Prevalence of Outcomes Excluding Aged 17 Years in Pre-Pandemic Cycle, NHANES 2015–2020

| | **Population** n (weighted %) | **Adequately Physically Active**[a] % (95% CI) | **"Eligible and Active"**[b] % (95% CI) |
|---|---|---|---|
| Total | | 69.3 (67.6, 70.9) | 34.9 (32.6, 37.3) |
| Gender | | | |
| Male | 2,800 (51.4) | 76.3 (73.6, 78.8) | 37.3 (33.5, 41.3) |
| Female | 2,924 (48.6) | 61.8 (59.6, 64.0) | 32.3 (29.2, 35.7) |
| Age, y | | | |
| 17–24 | 1,805 (27.9) | 75.9 (73.4, 78.3) | 44.1 (40.0, 48.3) |
| 25–29 | 1,094 (21.2) | 70.4 (66.7, 73.9) | 36.3 (31.8, 41.1) |
| 30–34 | 1,107 (20.6) | 69.3 (65.2, 73.0) | 32.4 (28.1, 37.0) |
| 35–42 | 1,718 (30.3) | 62.3 (59.0, 65.5) | 27.2 (24.3, 30.2) |
| Race/ethnicity | | | |
| NH White | 1,681 (55.3) | 72.7 (70.0, 75.3) | 39.1 (35.4, 42.9) |
| NH Black | 1,372 (12.7) | 66.7 (63.8, 69.5) | 29.6 (26.7, 32.8) |
| Hispanic | 1,588 (21.0) | 66.4 (63.2, 69.5) | 27.7 (24.7, 30.9) |
| NH Other | 1,083 (11.1) | 60.3 (55.7, 64.8) | 34.0 (30.9, 37.2) |
| Family Income[c] | | | |
| Low | 1,981 (28.5) | 67.4 (65.4, 69.4) | 32.4 (29.5, 35.3) |
| Moderate | 1,960 (38.9) | 70.4 (67.1, 73.5) | 32.4 (29.8, 35.0) |
| High | 1,099 (32.6) | 70.2 (66.2, 73.9) | 39.7 (33.7, 45.9) |

Values are weighted percentages based on non-pregnant persons aged 17–42 years, excluding those aged 17 years in the pre-pandemic (January 2017 – March 2020) cycle; unweighted n=5,724 for all variables except family income (n=5,040). Weighted percentages may not sum to 100% due to rounding.

NH, non-Hispanic.

[a]Defined as reporting ≥300 minutes/week of moderate-intensity physical activity or ≥150 minutes/week of vigorous-intensity physical activity or the equivalent combination, from all domains.

[b]Defined as body mass index 19.0–27.5 kg/m$^2$ and reporting ≥300 minutes/week of moderate-intensity physical activity or ≥150 minutes/week of vigorous-intensity physical activity or the equivalent combination, from all domains.

[c]Defined by the poverty income ratio: low, <150%; moderate, 150–400%; high, >400%.
